# Effects of lowering inspiratory oxygen fraction during microvascular decompression on postoperative gas exchange: A pre–post study

**DOI:** 10.1371/journal.pone.0206371

**Published:** 2018-11-14

**Authors:** Jungchan Park, Jeong Jin Min, So Jin Kim, Jin Hee Ahn, Keoungah Kim, Jong-Hwan Lee, Kwan Park, Ik Soo Chung

**Affiliations:** 1 Department of Anesthesiology and Pain Medicine, Samsung Medical Center, Sungkyunkwan University School of Medicine, Seoul, Korea; 2 Department of Neurosurgery, Samsung Medical Center, Sungkyunkwan University School of Medicine, Seoul, Korea; University of Virginia, UNITED STATES

## Abstract

**Background:**

Despite many previous studies, the optimal oxygen fraction during general anesthesia remains controversial. This study aimed to evaluate the effects of lowering intraoperative fraction of inspired oxygen on postoperative gas exchange in patients undergoing microvascular decompression (MVD).

**Methods:**

We conducted a pre-post study to compare postoperative gas exchange with different intraoperative oxygen fractions. From April 2010 to June 2017, 1456 consecutive patients who underwent MVD were enrolled. Starting in January 2014, routine oxygen fraction was lowered from 1.0 to 0.3 during anesthetic induction/awakening and from 0.5 to 0.3 during anesthetic maintenance. Postoperative gas exchange, presented as the minimum value of PaO2/FIO2 ratio within 48 hours, were compared along with adverse events.

**Results:**

Among 1456 patients, 623 (42.8%) patients were stratified into group H (high FIO2) and 833 (57.2%) patients into group L (low FIO2). Intraoperative positive end-expiratory pressure was used in 126 (15.1%) patients in group H and 90 (14.4%) patients in group L (p = 0.77).The minimum value of PaO2/ FIO2 ratio within 48 hours after surgery was significantly greater in the group L (226.13 vs. 323.12; p < 0.001) without increasing any adverse events.

**Conclusion:**

In patients undergoing MVD, lowering routine FIO2 and avoiding 100% O2 improved postoperative gas exchange.

## Introduction

Despite a 2016 World Health Organization (WHO) recommendation to use high intraoperative fraction of inspired oxygen (FIO2) to prevent surgical site infections [1], many anesthesiologists still use a high FIO2 only during anesthetic induction and awakening but a relatively low FIO2 during anesthetic maintenance. This protocol is used because of concerns that a high FIO2 will impair postoperative pulmonary function. In addition, a recent study has shown that a high intraoperative FIO2 was associated with major respiratory complications and with 30-day mortality in a dose-dependent manner [2].

Apart from the controversy of using high or low FIO2 during anesthetic maintenance, using 100% O2 during anesthetic induction and awakening could adversely influence the patient’s pulmonary function. Preoxygenation with 100% O2 leads to atelectasis within several minutes after induction of anesthesia [3,4] and using 100% O2 with airway suctioning during emergence and extubation also produces atelectasis [5–7]. However, to our best of knowledge, the effects of lowering FIO2 and avoiding 100% O2 entirely during general anesthesia including induction, maintenance and awakening, has never been studied.

Since January 2014, an anesthesiologist (I. S. Chung) in our hospital with more than 30 years of experience has avoided using 100% O2 entirely and lowered routine FIO2, from 1.0 to 0.3 during anesthetic induction/awakening and from 0.5 to 0.3 during anesthetic maintenance, in patients undergoing microvascular decompression (MVD). Because MVD is a stable procedure and and the corresponding patients are relatively healthy, we evaluated the postoperative outcomes with applying lower FIO2. Our hypothesis is that avoiding 100% O2 and lowering routine FIO2 throughout anesthesia would improve postoperative gas exchnage, which is presented as PaO2/FIO2 ratio, without increasing other complications.

## Methods

### Study population and data collection

This study used medical records of Samsung Medical Center located at Seoul, Korea. We compared postoperative gas exchange between the years when applying the relatively higher FIO2 and those when applying lower FIO2 during general anesthesia. From April 2010 to June 2017, a consecutive 1456 patients undergoing MVD, who were anesthesized by a single anesthesiologist (I. S. Chung) and performed also by a single surgeon (K. Park), were enrolled. FIO2 was lowered beginning in January 2014, therefore patients were divided into following two groups:group H (high FIO2 group, who underwent MVD before January 2014) and group L (low FIO2 group, who underwent MVD after January 2014). The electronic medical records of enrolled patients were collected and reviewed by a trained coordinator (J. Park) using a standardized form, and an independent investigator (J. J. Min) analyzed the data after deidentification.

This study was approved by the Institutional Review Board of Samsung Medical Center (IRB No. 2018-03-166) and conducted in accordance with the principles of the Declaration of Helsinki. The requirement for individual informed consent for this study was waived by the Institutional Review Board, as it was a retrospective study using electronic medical records.

### Anesthetic and postoperative management

In group H, FIO2 of 1.0 and 0.5 were used during anesthetic induction/awakening and maintenance, respectively. On the contrary, in group L, the routine FIO2 was lowered to 0.3 for the whole anesthetic procedures including induction/awakening and maintenance. Even when FIO2 was increased to treat hypoxemia, using 1.0 was entirely avoided in group L.

Except for FIO2, all anesthesia and ventilation were standardized in both groups as follows: after the patient arrived in the operating room, three-lead electrocardiography, pulse oximetry, and non-invasive arterial pressure were applied. Anesthesia was induced and maintained by propofol and remifentanil target-controlled infusions. After loss of consciousness, neuromuscular blockade was achieved with intravenous rocuronium (0.6 mg/kg) and a radial arterial catheter was inserted. After endotracheal intubation, ventilation was set at a tidal volume of 8 ml/kg of ideal body weight with a ventilator frequency adjusted to maintain normocarbia. In both groups, positive end-expiratory pressure (PEEP) was applied, if necessary. The tidal volume and PEEP were adjusted when peak airway pressure exceeded 25 cmH2O.

At the end of surgery, all patients were attempted to be extubated after confirming complete awakeness. In patients with successful extubation, supplemental oxygen was routinely delivered via facemask at 5 L/min or nasal cannula at 2 L/min. The flow rate was adjusted to avoid hypoxemia, if necessary. Postoperative evaluation included arterial blood gas analysis (ABGA) and chest plain film. An ABGA was performed upon arrival in the recovery room or intensive care unit using arterial line. Follow-up ABGAs or further radiographic evaluations were performed selectively in patients with dyspnea, a decrease in O2 saturation on pulse oximetry, or sudden hypotension.

### Study endpoints

The primary endpoint was the minimum value of PaO2/FIO2 ratio within 48 hours after surgery. Secondary endpoints were adverse events during recovery (delirium, nausea, vomiting, and delayed extubation > 1 hour), abnormal findings on chest film (atelectasis and pulmonary edema), surgical site infection, other infections (such as meningitis or upper airway infections), neurologic deficits (such as sensory change, palsy, or seizure), and other complications (such as postoperative acute kidney injury, hearing impairment, otorrhea, hematoma, or cerebrospinal fluid leakage). The peak levels of creatinine at 24 and 48 hours after surgery were also compared.

### Definitions

For PaO2/FIO2 ratio calculation, FIO2 was assumed to be 0.21 in the room air. When using oxygen delivery device such as facemask or nasal cannula, 0.3 was assumed for 5L/min with facemask and 2L/min with nasal cannula, and 4% of change was assumed per liter flow. Body mass index was defined as the body mass divided by the square of the body height and expressed in units of kg/m^2^. Chronic obstructive pulmonary disease was defined as any state of disease characterized by airflow limitation. A stable lung lesion on chest film included old tuberculosis, non-specific fibrosis, calcification and pulmonary nodule without symptom or change of size. Atelectasis was defined as the incomplete expansion or collapse of lung. Pulmonary edema was any condition associated with excessive fluid accumulation in the lung. All radiographic findings were confirmed by the department of radiology. Postoperative acute kidney injury was defined by the KDIGO (Kidney Disease Improving Global Outcomes) criteria using creatinine level [8].

### Statistical analysis

Continuous variables were compared with t-test or the Mann-Whitney test, and presented as mean ± standard deviation (SD). Chi-square or Fisher’s exact test were used for categorical variables. Linear regression analysis was used to compare PaO2/FIO2 ratio and creatinine level, and logistic regression analysis was used for other secondary endpoints. To reduce selection bias and adjust for confounding factors, propensity score matching was conducted on preoperative variables. After propensity score matching, an absolute standardized mean difference (SMD) < 10% was considered as an appropriate balance. In the propensity-matched population, a multiple linear regression analysis was used to adjust for intraoperative variables such as colloid use, crystalloid infusion, estimated blood loss, operative duration, and urinary output, to compare PaO2/FIO2 ratio and creatinine level. For secondary endpoints, multiple logistic regression analysis was conducted to adjust for intraoperative variables, and odds ratios (OR) with 95% confidence intervals (CI) were reported. All statistical analyses were performed wth SAS 9.4 (SAS Institute Inc., Cary, NC, USA). All tests were 2-tailed and p < 0.05 was considered statistically significant.

## Results

A total of 1456 patients were divided into two groups; 623 (42.8%) patients in group H and 833 (57.2%) patients in group L. In group L, FIO2 was 0.48±0.22 for induction and 0.37±0.07 for maintenance of anesthesia. Intraoperative PEEP was applied in 126 (15.1%) patients in group H and 90 (14.4%) patients in group L (p = 0.77). The maximal levels of PEEP were also not different between two groups (3.3 cmH2O vs. 3.1 cmH2O, p = 0.32). None of the patients, in either group had a major emergent hypoxemic event, such as failed tracheal intubation with oxygenation difficulty. The preoperative characteristics of both groups are summarized in Table 1. Patients in group L were older, had a higher incidence of underlying chronic obstructive pulmonary disease, and had higher preoperative levels of hemoglobin and albumin. Table 2 summarizes clinical outcomes of the entire population. The minimum value of PaO2/ FIO2 ratio within 48 hours after surgery was significantly greater in group L (226.13 vs. 323.12; p < 0.001) without any additional adverse events.

**Table 1:** Preoperative variables.

	Entire population				Propensity matched population		
	Group H (n = 623)	Group L (n = 833)	p-value	SMD	Group H (n = 619)	Group L (n = 619)	SMD
Inspired Oxygen Fraction							
Induction	1.0 (±0)	0.48 (±0.22)	< 0.0001		1.0 (±0)	0.48 (±0.22)	
Maintenance	0.5 (±0)	0.37 (±0.07)	< 0.0001		1.0 (±0)	0.37 (±0.06)	
Male	194 (31.4)	249 (29.9)	0.609	-2.7	191 (30.9)	191 (30.9)	0
Age	51.6 (±11.39)	53.3 (±10.57)	0.005	16.1	51.6 (±11.33)	52.4 (±10.76)	7.4
BMI (kg/m^2^)	24.41 (±3.53)	24.23 (±3.24)	0.576	-5.4	24.41 (±3.52)	24.27 (±3.31)	-4.3
Previous Conditions							
COPD	6 (1.0)	30 (3.6)	0.001	14.2	6 (1.0)	6 (1.0)	0
Tuberculosis	5 (0.8)	10 (1.2)	0.457	3.7	5 (0.8)	6 (1.0)	1.5
Smoking	3 (0.5)	8 (1.0)	0.37	4.9	3 (0.5)	4 (0.7)	1.7
Hypertension	169 (27.1)	179 (21.5)	0.013	-13.7	167 (27.0)	143 (23.1)	-9.4
Diabetes	25 (4.0)	35 (4.2)	0.858	0.9	25 (4.0)	26 (4.2)	0.8
CAD	8 (1.3)	7 (0.8)	0.407	-4.9	8 (1.3)	3 (0.5)	-8.8
Chest Plain Film							
Active Lesion	5 (0.8)	9 (1.1)	0.591	2.7	5 (0.8)	5 (0.8)	0
Stable Lesion	11 (1.8)	25 (3.0)	0.133	7.2	10 (1.6)	17 (2.8)	6.6
Blood Tests							
Hemoglobin (g/dl)	13.38 (±1.45)	13.19 (±1.45)	0.012	-13	13.37 (±1.44)	13.25 (±1.48)	-8.6
Albumin (g/dl)	4.35 (±0.28)	4.33 (±0.28)	0.043	-8.9	4.35 (±0.28)	4.34 (±0.29)	-6.3
Creatinine (mg/dl)	0.75 (±0.17)	0.75 (±0.16)	0.458	0.4	0.75 (±0.17)	0.75 (±0.16)	1.2

Values are n (%) or mean (±SD)

SMD, standard mean difference; BMI, body mass index; COPD, chronic obstructive pulmonary disease; CAD, coronary arterial disease

**Table 2:** Clinical outcomes of the entire population.

	Group H (n = 623)	Group L (n = 833)	Unadjusted Odds Ratio (95% CI)	p-value
PaO2/FIO2	226.13 (±125.32)	323.12 (±251.90)		<0.0001
Any Postoperative Adverse Event	239 (38.4)	305 (36.6)	0.93 (0.75–1.15)	0.495
Adverse Event during Recovery	217 (34.8)	283 (34.0)	0.96 (0.77–1.20)	0.733
Delirium	13 (2.1)	21 (2.5)	1.21 (0.60–2.44)	0.588
Nausea	170 (27.3)	230 (27.6)	1.02 (0.81–1.28)	0.891
Vomiting	115 (18.5)	127 (15.3)	0.80 (0.60–1.05)	0.104
Delayed Extubation	50 (8.03)	48 (5.76)	0.70 (0.47–1.06)	0.089
Postoperative Acute Kidney Injury	4 (0.6)	6 (0.7)	1.12 (0.32–4.0)	0.858
Maximal Creatinine (mg/dl)				
Within 24 hours	0.88 (±0.34)	0.96 (±0.30)		<0.0001
Within 48 hours	0.91 (±0.30)	0.99 (±0.12)		<0.0001
Atelectasis	4 (0.6)	0		
Pulmonary Edema	0	3 (0.4)		
Surgical Site Infection	9 (1.4)	15 (1.8)	1.25 (0.54–2.88)	0.598
Other Infection	7 (1.2)	4 (0.5)	0.43 (0.12–1.46)	0.425
Neurologic Deficit	5 (0.8)	0		
Other Adverse Event	27 (4.3)	23 (2.8)	0.63 (0.36–1.10)	0.627

Values are n (%) or mean (±SD)

Other adverse events included hearing impairment, otorrhea, hematoma, and cerebrospinal fluid leakage

A total of 619 data pairs were generated by 1:1 individual matching without replacement. A propensity score for all preoperative variables were used for matching, and an absolute SMD < 10% suggested an appropriate balance between matched groups (Table 1). Intraoperative variables of the propensity-matched population are present in Table 3. Intraoperative variables were adjusted to compare clinical outcomes in the propensity-matched population. The minimum value of PaO2/ FIO2 ratio within 48 hours after surgery was significantly greater in group L (226.37 vs. 330.87; p < 0.001). The peak creatinine levels at 24 and 48 hours after surgery were higher in group L (0.68 vs. 0.72; p < 0.001, 0.70 vs. 0.73; p < 0.001, respectively), but the risk of postoperative acute kidnry injury was not significantly different (0.6% vs. 0.8%; OR 2.51; CI 0.56–11.30; p = 0.232). The incidence of vomiting was significantly lower in group L (18.4% vs. 15.0%; OR 1.54; CI 1.06–2.25; p = 0.02) (Table 4). No other complications differed significantly between groups. Minimal dataset is provided in S1 Dataset.

**Table 3:** Intraoperative variables in the propensity-matched population.

	Group H (n = 619)	Group L (n = 619)	p-value
Colloid use (%)	478 (76.6)	300 (35.9)	<0.0001
Crystalloid infusion (ml)	1159 (±318)	938 (±230)	<0.0001
EBL (ml)	237 (±116)	147 (±119)	<0.0001
Operative duration (min)	120 (±23)	102 (±18)	<0.0001
Urine output (ml)	320 (±247)	329 (±212)	0.479

Values are n (%) or mean (±SD)

EBL, estimated blood loss

**Table 4:** Clinical outcomes in the propensity-matched population.

	Group H (n = 619)	Group L (n = 619)	Unadjusted Odds Ratio (95% CI)	p-value	Adjusted Odds Ratio (95% CI)	p-value
PaO2/FIO2	226.37 (±124.45)	330.87 (±254.46)		<0.0001		<0.0001
Any Postoperative Adverse Event	236 (38.1)	234 (37.8)	0.99 (0.78–1.24)	0.907	1.05 (0.79–1.41)	0.726
Adverse Event during Recovery	214 (34.6)	217 (35.1)	1.02 (0.81–1.29)	0.858	1.02 (0.76–1.37)	0.887
Delirium	13 (2.1)	17 (2.8)	1.32 (0.63–2.73)	0.461	2.13 (0.82–5.11)	0.116
Nausea	168 (27.1)	175 (28.3)	1.06 (0.83–1.36)	0.657	0.94 (0.68–1.28)	0.675
Vomiting	114 (18.4)	93 (15.0)	0.78 (0.58–1.01)	0.11	0.65 (0.44–0.95)	0.025
Delayed Extubation	49 (7.9)	37 (6.0)	0.74 (0.48–1.15)	0.181	0.93 (0.53–1.62)	0.792
Postoperative Acute Kidney Injury	4 (0.6)	5 (0.8)	1.25 (0.34–4.69)	0.738	2.51 (0.56–11.30)	0.232
Maximal Creatinine (mg/dl)						
Within 24 hours	0.68 (±0.18)	0.72 (±0.17)		<0.0001		<0.0001
Within 48 hours	0.70 (±0.18)	0.73 (±0.17)		<0.0001		<0.0001
Atelectasis	4 (0.7)	0				
Pulmonary Edema	0	3 (0.5)				
Surgical Site Infection	9 (1.5)	11 (1.8)	1.23 (0.51–2.98)	0.653	1.97 (0.65–6.01)	0.231
Other infection	7 (1.1)	3 (0.5)	0.43 (0.11–1.65)	0.218	0.65 (0.13–3.16)	0.591
Neurologic Deficit	5 (0.8)	0				
Other Adverse Events	27 (4.4)	18 (2.9)	0.66 (0.36–1.21)	0.175	0.89 (0.42–1.89)	0.762

Values are n (%) or mean (±SD)

Covariates included intraoperative parameters (colloid use, crystalloid infusion, estimated blood loss, operative duration, urine output)

Other adverse events included hearing impairment, otorrhea, hematoma, and cerebrospinal fluid leakage

## Discussion

Our study showed that lowering routine FIO2 from 1.0 to 0.3 during anesthetic induction and awakening and from 0.5 to 0.3 during anesthetic maintenance improved the postoperative PaO2/FIO2 ratio in patients undergoing MVD. Our result suggests that avoiding 100% O2 and maintaining low FIO2 during general anesthesia might be associated with the improvement of postoperative gas exchange.

Despite many previous studies and even a recent WHO guideline, the optimal O2 concentration during general anesthesia still remains controversial [9,10]. Therefore, in daily anesthetic practice, setting FIO2 appears to be determined based on personal preference or routine clinical practice of each hospitals rather than on the evidence-based guidelines [11]. During anesthetic induction and awakening, 100% O2 has been widely used in daily anesthetic practice because high FIO2 expands the time periods for developing unacceptable desaturation when occurring unexpected difficulty of airway maintenance [12]. However, using 100% O2 has been shown to induce atelectasis within 5 minutes [12], and postoperative atelectasis has a definitely harmful effect on the patients’ outcomes [13]. In this study, all patients were closely monitored by an experienced anesthesiologist who was prepared for the treatment of difficult airway, and no patients showed unanticipated hypoxemic event to require an emergent intervention. Therefore, considering the harmful effect on the patient’s postoperative outcome, using 100% O2 instead of just high FIO2 during those periods might be questionable in case that the experienced anesthesiologists for airway management is present. In 2014, Habre W and Peták F have recommended the use of 80% O2 during anesthetic induction and awakening in their recent review article based on taking into consideration the minimum risk/benefit ratio [10].

The optimal O2 concentration during anesthetic maintenance is much more highly debated than that during anesthetic induction and awakening. Traditionally, the use of high O2 concentration during surgery has been suggested to reduce the risk of surgical site infection and postoperative nausea and vomiting [14,15]. In addition, despite the evidences of adeverse effects related with high O2 concentration on the pulmonary system [16], previous studies have failed to demonstrate the exact relationship between the use of intraoperative high FIO2 and an increase in postoperative pulmonary complications [17,18]. Therefore, in 2016, the WHO has recommended the use of 80% FIO2 in all intubated patients during surgery and the postoperative use of high-flow facial mask for several hours [1]. However, based on the current literatures, it remains very controversial whether O2 supplement reduces surgical site infection. Several previous studies have shown the beneficial effect of supplemental O_2_ on surgical site infection [19–22] but the other studies have not [14,23–25]. In addition, a recent Cochrane systematic review in which included both the studies used in an WHO guideline and the more recent randomized trials, has suggested that the supporting evidence of using routinely high FIO2 during anesthesia is not sufficient [26].

A recent study has suggested that intraoperative FIO2 is associated with postoperarive pulmonary complications in a dose-dependent manner [2]. In this study, routine FIO2 was lowered from 1.0 to 0.3 for anesthetic induction and 0.5 to 0.3 during maintenance, and FIO2 1.0 was entirely avoided even when higher FIO2 was applied to treat hypoxemia. Our hypothesis was that these changes had improved postoperative gas exchange, presented as PaO2/FIO2 ratio [27]. An improved gas exchange may be explained by the occurrence and amount of atelectasis which leads to intrapulmonary shunt. When high fraction oxygen is rapidly absorbed into closed airways during general anesthesia, atelectasis and shunt occur causing gas exchange abnormality [28]. However, in the absence of serial measurements, whether improved PaO2/FIO2 ratio is directly associated with postoperative pulmonary function remains unconfirmed in this study.

Interestingly, the incidence of postoperative vomiting decreased in the low FIO2 group. That result dose not correlate with previous data which have suggested the O2 supplement can decrease the incidence of postoperative nasea and vomiting [29,30]. However, the neurosurgical procedure is a high-risk procedure for nausea and vomiting [31]. Therefore, the incidence of postoperative vomiting might be less related to intraoperative FIO2, but more to surgery itself.

This study has several limitations. First, our results were from a retrospective analysis. Therefore, it would be possible that unmeasured confounding factors would not be adjusted even after propensity score matching. In particular, different FIO2 at ABGA measurement might have biased the results because PaO_2_/ FIO2 ratio is highly dependent on FIO2. In addition, due to the absence of detailed protocol for postoperative care, different indications for supportive care and follow-up evaluation may have been applied. Second, except the improvement of PaO_2_/ FIO2 ratio, the incidences of the other pulmonary complications showed no difference between two groups. However, the incidence of pulmonary complications in this study would be too low to compare the exact relationship between the lowering routine FIO2 and the postoperative pulmonary complications. Lastly, patients with severe comorbidities were not enrolled in this study. Therefore, it would be hard to conclude whether lowering routine FIO2 has a beneficial effect among high-risk patients.

In conclusion, in the patients undergoing MVD, lowering intraoperative FIO2 and avoiding 100% O2 during anesthetic induction and awakening may improve the postoperative gas exchange. However, the exact relationship between intraoperative FIO2 and postoperative outcomes remains to be evaluated. This study does not comment upon pulmonary function but only upon gas exchange and is not able to conclude upon complications.

## Supporting information

S1 Dataset Minimal dataset. (XLSX)

## Abbreviations and Acronyms

WHO: World Health Organization
FIO2: fraction of inspired oxygen
MVD: microvascular decompression
PEEP: positive end-expiratory pressure
ABGA: arterial blood gas analysis
